# A Rapid Detection Method for Fungal Spores from Greenhouse Crops Based on CMOS Image Sensors and Diffraction Fingerprint Feature Processing

**DOI:** 10.3390/jof8040374

**Published:** 2022-04-06

**Authors:** Yafei Wang, Hanping Mao, Guilin Xu, Xiaodong Zhang, Yakun Zhang

**Affiliations:** 1 Key Laboratory of Modern Agricultural Equipment and Technology, Ministry of Education, Jiangsu University, Zhenjiang 212013, China; 2111916018@stmail.ujs.edu.cn (Y.W.); 2212016052@stmail.ujs.edu.cn (G.X.); 1000001703@stmail.ujs.edu.cn (X.Z.); zhangyk@haust.edu.cn (Y.Z.); 2 School of Agricultural Engineering, Jiangsu University, Zhenjiang 212013, China

**Keywords:** greenhouse crop, pathogenic spore, CMOS image sensor, diffraction image processing, SVM

## Abstract

The detection and control of fungal spores in greenhouse crops are important for stabilizing and increasing crop yield. At present, the detection of fungal spores mainly adopts the method of combining portable volumetric spore traps and microscope image processing. This method is problematic as it is limited by the small field of view of the microscope and has low efficiency. This study proposes a rapid detection method for fungal spores from greenhouse crops based on CMOS image sensors and diffraction fingerprint feature processing. We built a diffraction fingerprint image acquisition system for fungal spores of greenhouse crops and collected diffraction fingerprint images of three kinds of fungal spores. A total of 13 diffraction fingerprint features were selected for the classification of fungal spores. These 13 characteristic values were divided into 3 categories, main bright fringe, main dark fringe, and center fringe. Then, these three features were calculated to obtain the Peak to Center ratio (PCR), Valley to Center ratio, and Peak to Valley ratio (PVR). Based on these features, logistics regression (LR), K nearest neighbor (KNN), random forest (RF), and support vector machine (SVM) classification models were built. The test results show that the SVM model has a better overall classification performance than the LR, KNN, and RF models. The average accuracy rate of the recognition of three kinds of fungal spores from greenhouse crops under the SVM model was 92.72%, while the accuracy rates of the LR, KNN, and RF models were 84.97%, 87.44%, and 88.72%, respectively. The F1-Score value of the SVM model was higher, and the overall average value reached 89.41%, which was 11.12%, 7.18%, and 5.57% higher than the LR, KNN, and RF models, respectively. Therefore, the method proposed in this study can be used for the remote identification of three fungal spores which can provide a reference for the identification of fungal spores in greenhouse crops and has the advantages of low cost and portability.

## 1. Introduction

With the acceleration of urbanization, people’s demand for a “vegetable basket” project is increasing. In recent years, China’s vegetable industry has developed rapidly, and it now has a greenhouse cultivation area of more than 4 million hectares [1,2]. In addition, greenhouse cultivation in China is dominated by two kinds of vegetables, tomatoes, and cucumbers [1,3]. Greenhouse vegetable production is usually in the semi-closed state of high temperature, high humidity, and high evaporation. There is a serious problem with fungal infections in greenhouse production [4]. In particular, cucumber downy mildew (*Pseudoperonospora cubensis*), cucumber powdery mildew (*Podosphaera xanthii*), and tomato gray mold (*Botrytis cinerea*). The severity of these diseases is associated with the dynamic destruction of the assimilation area of leaves and fruit. At the same time, the pathogens form numerous spores that are dispersed through the air, further infecting tomato and cucumber plants [5]. These fungal diseases can cause diseases on the fruit, stems, and leaves of cucumbers and tomatoes. In severe cases, crop yields can be reduced, with a yield loss of 20–50%, or even no harvest [6,7,8]. Therefore, timely detection and control of greenhouse fungal pathogens are important in stabilizing and improving crop yield [9,10].

Currently, direct and indirect disease identification methods are used for plant disease detection, such as enzyme-linked immune-sorbent assay, immunofluorescence, fluorescence in situ hybridization, fluorescence imaging, and hyperspectral technology [11,12,13,14,15]. Although these methods are effective, they are more difficult for ordinary farmers to implement. In recent years, with the rapid development of deep learning in the field of computer vision, the recognition of plant diseases based on leaf disease images has attracted the attention of related scholars [16,17,18,19]. For example, Zhang et al. [20] completed a study to identify cucumber diseases in greenhouses under natural and complex conditions. They collected cucumber powdery mildew, downy mildew, and healthy leaves. They used the pre-trained EfficientNet-B0-B7 model to transfer learning and constructed the classification model of powdery mildew, downy mildew, the combination of powdery mildew and downy mildew, and healthy leaves to realize the accurate classification of cucumber diseases. Liu et al. [21] investigated improving the detection efficiency of identifying disease cucumber leaves in different images with complex backgrounds. They proposed an efficient detection model (EFDet), which mainly consisted of an efficient backbone network, a feature fusion module, and a predictor. They collected three categories of cucumber leaves including downy mildew, bacterial angular spot, and healthy leaves to construct the cucumber disease dataset. Experimental results revealed that EFDet has strong robustness for identifying disease cucumber leaves in complex environments. Zhou et al. [22] studied the identification of common invasive diseases in tomatoes and cucumbers. They constructed a disease identification model based on an “image-text” multimodal collaborative representation and knowledge assistance (ITK-Net). The proposed model achieved an identification accuracy, precision, sensitivity, and specificity of 99.63%, 99%, 99.07%, and 99.78%, respectively on a dataset composed of “imagetext” pairs. Although the plant disease detection methods based on deep learning can provide accurate, fast, and efficient disease diagnosis [23,24], these diagnostic techniques cannot detect crop diseases before they become prevalent, only when the diseases occur and by this time the optimal window for prevention and control has been missed [25,26].

In recent years, in order to achieve early detection of crop diseases, scholars have used portable spore traps to capture airborne disease spores and then combined these with image processing methods for early detection of airborne disease spores in crops. For example, in order to achieve quantitative monitoring of airborne spores, Lei et al. [27] proposed an algorithm for the automatic detection and counting of spores based on digital image processing using the K-means clustering algorithm, image preprocessing, the identification of touching spores based on their shape factor and area, and touching spore contour segmentation based on concavity and contour segment merging. Wang et al. [28] investigated achieving the identification of spores of airborne diseases in greenhouse crops. Their research focused on cucumber downy mildew spores, cucumber powdery mildew spores, and tomato gray mold spores. The color, shape, and texture of the three disease spores were extracted. Based on these features, LR, KNN, RF, and SVM classification models were established. The average accuracy of SVM, LR, KNN, and RF models were 94.36%, 90.13%, 89.37%, and 89.23%, respectively. A study by Li et al. [29] investigated automated counting of the urediospores of *Puccinias triiformis f.* sp. *tritic*. Transparent tapes, glass slides with vaseline, and Eppendorf centrifuge tubes were used to simulate the capture of the urediospores of *Puccinias triiformis f.* sp. *Tritic*, and images of the spores were obtained using microscope photography. The satisfactory results for counting the trapped spores were obtained after processing the spore images acquired by using the three kinds of simulation methods. The average counting accuracy for the urediospores of *P. striiformis f.* sp. *tritici* trapped on transparent tapes, glass slides with vaseline, and in Eppendorf centrifuge tubes was 98.5%, 98.7%, and 99.9%, respectively. The average counting accuracy for the urediospores of *P. striiformis f.* sp. *tritici* mixed with the conidia of *Blumeria graminis f.* sp. *tritici*, which can cause wheat powdery mildew, was 99.8%. Qi et al. [30] investigated the automatic detection and counting of the spores of rice blast. The spore images of the rice blast were obtained using a microscopic imaging system. The results show that among the 100 microscopic image samples tested, the average accuracy of spore detection was 98.5%. Although the above method can realize the detection of disease spores, due to the small size and the large number of spores, and the small field of view of the microscope, large counting errors occur easily.

With the development of sensor technology, lens-less CMOS image sensors are favored by related researchers due to their large imaging field of view and low cost. For example, Wang et al. [31] collected diffraction images of disease spores using a CMOS image sensor to count tomato gray mold spores based on the diffraction reconstruction method. Yang et al. [32] proposed a rapid detection and identification method of rice blast spores based on the diffraction fingerprint texture of the spores of crop pathogens. In summary, the method based on CMOS image sensors and diffraction fingerprint image processing can be used for the detection of airborne disease spores in greenhouses.

Hence, in this study, in order to achieve the rapid detection of the fungal spores of greenhouse-crop airborne diseases, a rapid detection method based on CMOS image sensors and diffraction fingerprint feature processing is proposed. The diffraction fingerprint image acquisition system for airborne disease spores of greenhouse crops was built. Diffraction fingerprint images of three kinds of fungal spores were collected. The diffraction fingerprint feature of fungal spores was extracted and the classification model was established. The classification and detection of greenhouse crop fungal spores were realized.

## 2. Materials and Methods

### 2.1. Fungal Spore Sample Collection and Parameter Measurement

**Figure 1:**
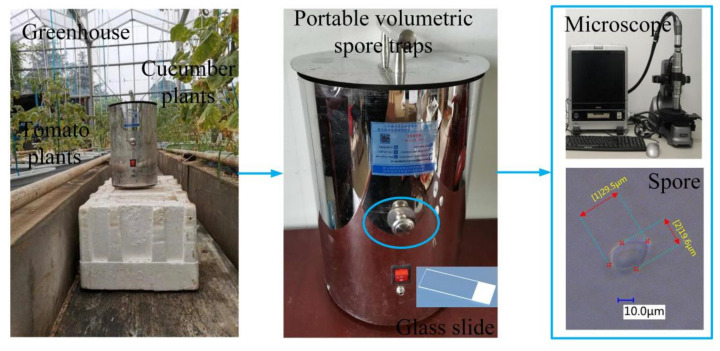
Process of collecting fungal spores and measuring parameters.

This study was conducted in a Venlo-type greenhouse at Jiangsu University, Zhenjiang, China. The greenhouse is east–west aligned, with a length of 100 m, a width of 40 m, a shoulder height of 2.4 m, and a span of 2.4 m. In order to collect fungal spore samples of airborne diseases, cucumber and tomato plants were planted in the greenhouse from April 2020 to September 2021 (Figure 1). The cultivated tomato hybrid was ‘Zhefen 202’. The cultivated cucumber hybrid was ‘Jinyou No. 1’. *P. cubensis* and *P. xanthii* occurred in cucumber plants and *B. cinerea* occurred in tomato plants. We collected cucumber downy mildew spores and cucumber powdery mildew spores from the leaves of cucumber plants, and tomato gray mold spores from the leaves of tomato plants. We then prepared the spore suspension and re-inoculated the cucumber and tomato plants until the cucumber plants developed cucumber downy mildew and powdery mildew and the tomato plants developed tomato gray mold. To obtain diffraction images of pathogenic spores, first a portable volumetric spore trap (OK-BZ1, Zhengzhou Oukeqi Instrument Manufacturing Co., Ltd., Zhengzhou, China) was used to capture pathogenic spores in the air. Then, a sample of the pathogenic spores was brought into the laboratory, where they were observed and identified using a microscope with an extended depth of field (VHX-900F, made by KEYENCE Co., Osaka, Japan). The fungal spores were then measured using this microscope. *B. cinerea* spores are almost oval and have a size distribution of 19.3(11.4–26.7) × 11.7(8.3–14.5) μm, *P. cubensis* spores are lemon shape and the size distribution is 30.6(21.1–39.8) × 20.5(13.8–23.6) µm, and *P. xanthii* spores are cylindrical and the size distribution is 35.4 (30.2–39.5) × 14.2 (7.3–22.2) µm. Finally, the slides with pathogenic spores were placed in a diffraction device to collect their diffraction fingerprints. All experiments were conducted in the Key Laboratory of Modern Agricultural Equipment and Technology of the Ministry of Education of Jiangsu University, China.

### 2.2. Construction of the Fungal Spore Diffraction Fingerprint Collection System

**Figure 2:**
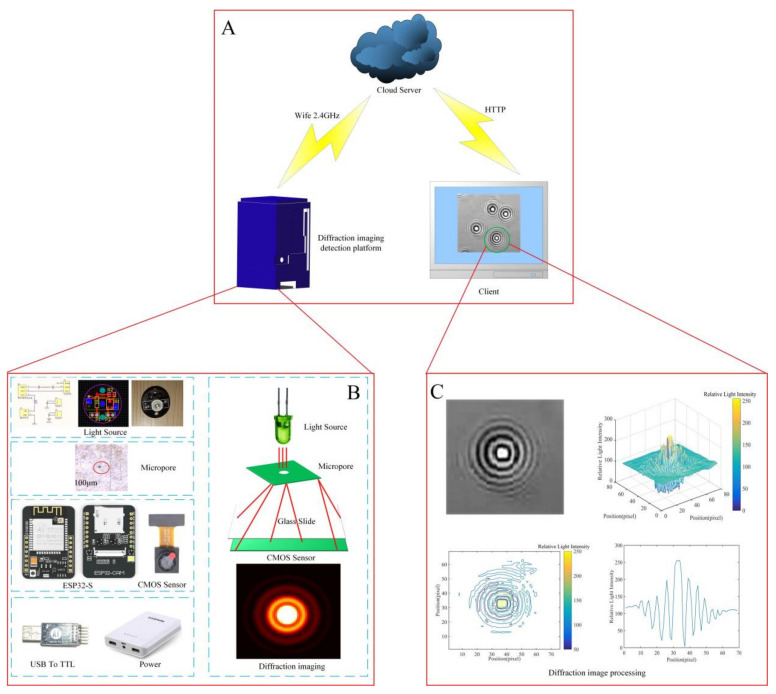
The fungal spores diffraction fingerprint collection system, (**A**) diffraction fingerprint collection system architecture, (**B**) diffraction fingerprint image detection platform, and (**C**) diffraction fingerprint image processing.

Traditional micro-optical imaging technology refers to the technology in which the visible light transmitted through or reflected from the sample passes through one or more lenses to obtain a magnified image of a tiny sample. The size of the field of view of the image that can be presented is determined by the number of fields of view of the eyepiece. The size of plant-fungal spores is generally a few micrometers to tens of micrometers [3,28,33]. When observing fungal spores with traditional optical microscopes, a larger magnification is required which will lead to a decrease in the number of actual fungal spores observed. In addition, traditional optical microscopes can only collect image information of fungal spores but cannot reflect the characteristics of fungal spores themselves. Diffraction refers to the phenomenon that light encounters opaque or transparent obstacles or micropores (narrow slits) in the propagation path, bypasses the obstacles, and deviates from the straight line. Under the appropriate circumstances, any wave has the inherent properties of diffraction and the degree of wave diffraction varies in different situations. If the obstacle has multiple densely distributed pores, it will cause a more complicated diffraction intensity distribution pattern. The size, shape, and surface texture characteristics of different fungal spores are varied. The light intensity distribution of light and dark stripes generated during diffraction can reflect the characteristics of fungal spores. Therefore, in order to collect the diffraction fingerprint image information of fungal spores, the fungal spore diffraction fingerprint acquisition system was established as shown in Figure 2.

The system is mainly composed of three parts: the fungal spores diffraction fingerprint image detection platform, the cloud server, and the computer. The cloud server can be accessed through the computer web page. The fungal spores diffraction image detection platform is mainly composed of a LED light source, a micropore, a CMOS image sensor, a power supply, and a USB to TTL, ESP32-S development board. Traditional diffraction image shooting mainly uses a laser with good coherence as the light source, but this type of laser is expensive, has more peripheral drivers, and the portability is very poor. The laser light path is complex, and it needs to be collimated and expanded by a lens, and then coupled with a micropore, which requires high component processing and assembly technology. The laser has high energy and it is easy to damage the sample. The time coherence of the laser is high, which will produce speckle noise on the holographic image and affect the accuracy of observational results. Therefore, this study used a common LED lamp as the light source module, which was low cost, small volume, had low-energy consumption, and also avoided damage to the sample. The wavelength of the LED light source was 450–465 nm. Due to the poor coherence of the LED lamp itself, a micropore was needed to produce the partially coherent light coupled with it. Micropores were 100 μm in diameter and were installed directly under the LED lamp. A 2-million-pixel CMOS (LAAF2569) image sensor (Shenzhen Cool Vision Technology Co., Ltd., Shenzhen, China) was used. The resolution of the captured image was 1280 × 1024 dpi. The external dimensions were 8 mm × 8 mm × 5.40 mm. The working temperature was −20–70 °C. The working voltage was 1.7−3.3 V. The CMOS sensor was installed 45mm directly below the micropore. The size of the ESP32-S development board was 27 × 40.5 × 4.5 (±0.2) mm. The working voltage was 4.75–5.25 V. The working temperature was −20–70 °C. The spectrum range was 2400–2483.5 MHz. It was able to use wireless WiFi networking and supported 802.11 b/g/n/e/i protocols. Image data could also be transmitted via Bluetooth, supporting Bluetooth 4.2 BR/EDR and BLE standards. The diffraction image detection platform of the disease spores could transmit the collected disease spore diffraction fingerprint image to the cloud server through the WiFi. Bafa Cloud was used in this system. The staff could process and observe the diffraction fingerprint image of the disease spore for the client. The system realized the independent operation of remote photographing, sampling, and detection of fungal spores’ diffraction fingerprint images.

### 2.3. Fungal Spore Diffraction Fingerprint Image Processing

**Figure 3:**
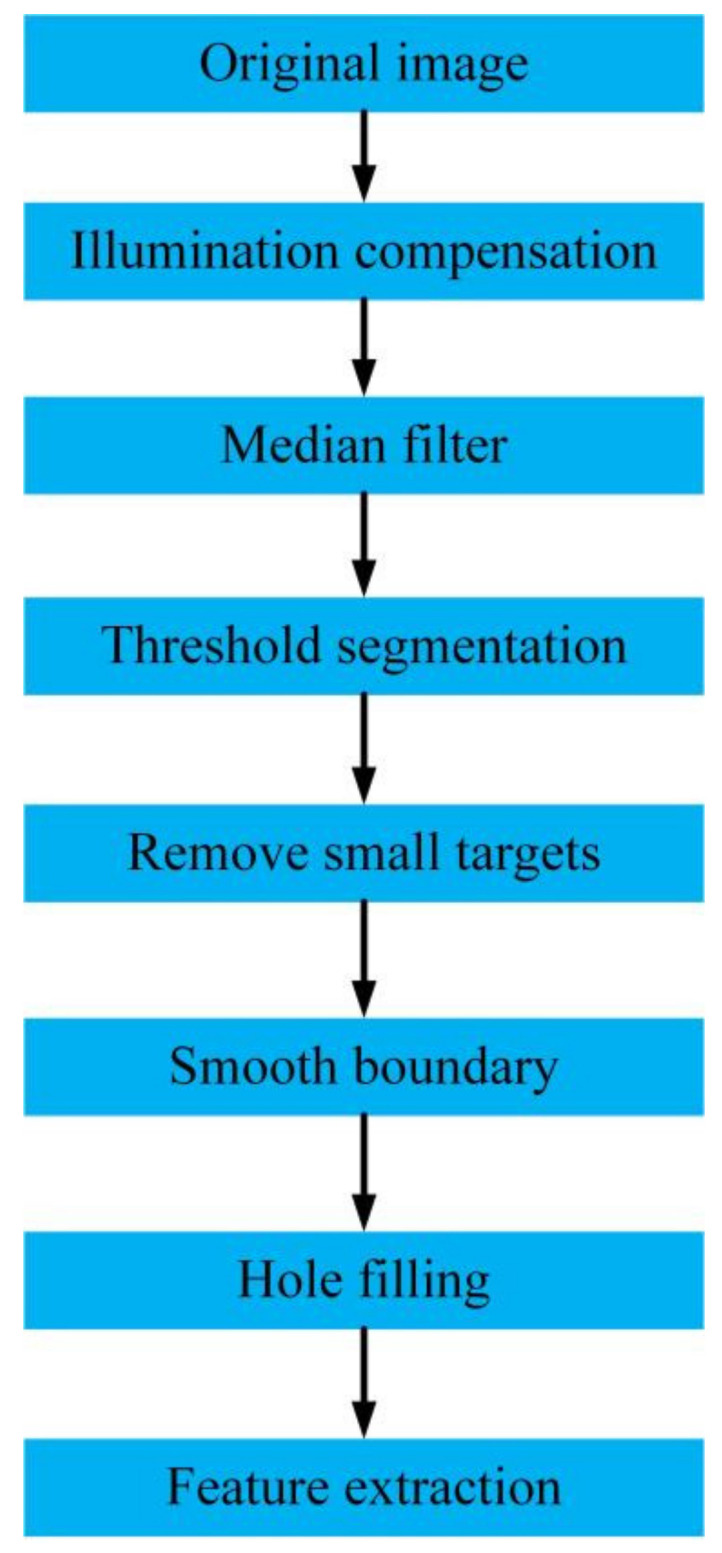
Diffraction fingerprint image pre-processing process.

The fungal spore diffraction fingerprint collection system is easily affected by the external environment where it works. Diffraction fingerprint images of fungal spores may have uneven brightness and salt and pepper noise. Therefore, in order to reduce the unusable information of the diffracted fingerprint image and retain valid information, the diffractive fingerprint image needs to be preprocessed. The preprocessing flow of the diffraction fingerprint image of fungal spores is shown in Figure 3.

First, in order to solve the problems of underexposure and uneven illumination that may exist in the fungal spore diffraction fingerprint collection system a new two-dimensional Gamma function was constructed to correct the brightness of the original image of the collected fungal spore diffraction fingerprints. The expression of the two-dimensional Gamma function is as follows [33]:

(1) $$O(x,y)=255{\left(\frac{F(x,y)}{255}\right)}^{\gamma }$$

(2) $$\gamma ={\left(\frac{1}{2}\right)}^{\frac{m-I(x,y)}{m}}$$

where *O*(*x*, *y*) is the brightness value of the corrected output image, *F*(*x*, *y*) is the original input image, *γ* is an exponential value used for brightness enhancement, which contains the characteristics of the illumination component of the image, *I*(*x*, *y*) is the extracted light component, and *m* is the mean value of the brightness of the illumination component.

Second, in order to preserve the detailed features of the diffracted fingerprint image of the fungal spores, it was necessary to suppress the noise of the images. Median filtering is a kind of non-linear filtering. It has an excellent effect when dealing with impulse noise and salt and pepper noise, and it can effectively protect the edge information of the image. Therefore, this study used median filtering to reduce the salt and pepper noise of the diffracted fingerprint images of fungal spores.

Third, the maximum between-cluster variance method was used for automatic threshold segmentation of fungal spore diffraction fingerprint images. Then, the diffracted fingerprint image was smoothed and filled with holes through morphological operations such as expansion and erosion, and other interfering targets were removed to obtain the target image.

### 2.4. Relative Light Intensity Distribution of Diffraction Fingerprint Image

The diffraction imaging system in this research was designed according to the Huygens–Fresnel principle. Diffraction images satisfy the relationship between Fourier transform and inverse transform. The complex amplitude of point *P* on the diffraction image can be expressed as [31]:

(3) $$U(P)=k\iint U_{0}(Q)\;F(\theta_{0},\theta )\frac{e^{ikr}}{r}dxdy=\left|U(P)\right|\cdot e^{iap}$$

where |*U*(*P*)| is used to represent amplitude information and *e^iaq^* is used to represent phase information. A CMOS sensor was used to take diffraction images of fungal spores. The square of the light intensity information amplitude of the diffraction image is:

(4) $$I(P)={\left|U(P)\right|}^{2}$$

Due to the inevitable phase loss phenomenon in the diffraction imaging process, it is necessary to perform phase recovery processing on the diffraction image of fungal spores. In this study, sampling theory and iterative algorithms were used for phase recovery of fungal spores’ diffraction images.

### 2.5. Feature Extraction of Fungal Spore Diffraction Fingerprint

**Figure 4:**
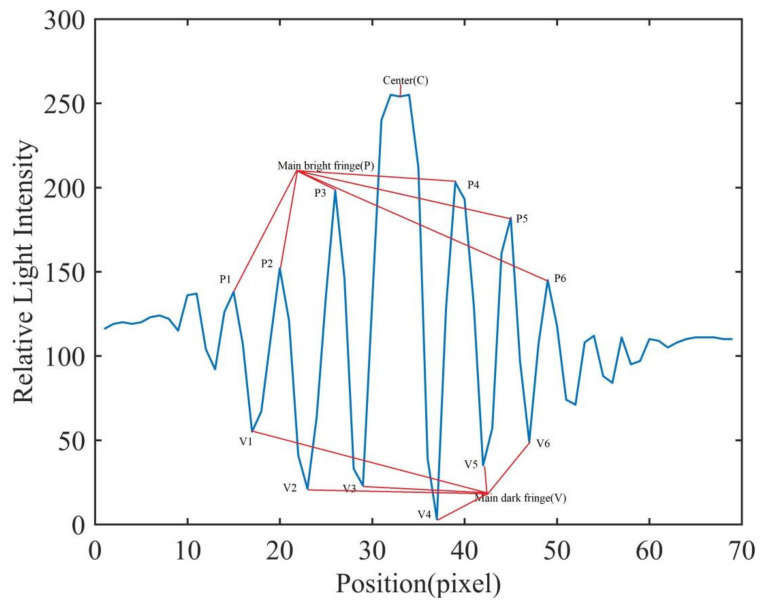
The relative light intensity distribution of fungal spore diffraction fingerprints.

In the diffracted fingerprint image of fungal spores, the light intensity distribution of the diffracted fingerprint area is closely related to the type, size, geometric shape, light absorption, and other factors of the spore. It can be seen from the relative light intensity distribution diagram of the spore diffraction fingerprint (Figure 4) that the diffraction fingerprint was clearest, the visibility is the highest, and the energy is the most concentrated near the center. The interference at the edges decayed rapidly and visibility decreased. The pixel distribution in the area close to the center of the diffracted fingerprint contributed the most to the characteristic value, and the main fringe was more valuable. Therefore, this study mainly chooses the main stripes of the spore diffraction fingerprint as the spore diffraction fingerprint feature for the classification of fungal spores. As shown in Figure 4, a total of 13 diffraction fingerprint features were selected for the classification of fungal spores. These 13 characteristic values were divided into 3 categories, main bright fringe, main dark fringe, and center fringe. Then, these three categories were calculated to obtain Peak to Center ratio (PCR), Valley to Center ratio, and Peak to Valley ratio (PVR). The formulas for calculating these three parameters are as follows:

(5) $$\mathrm{PCR}=\frac{{\mathrm{AP}}_{i}}{C}\;\;\left(i=1,2,3,4,5,6\right)$$

(6) $$\mathrm{VCR}=\frac{AV_{j}}{C}\;\;\left(j=1,2,3,4,5,6\right)$$

(7) $$\mathrm{PVR}=\frac{{\mathrm{AP}}_{i}}{{\mathrm{AV}}_{j}}\;\;\left(i=1,2,3,4,5,6;\;j=1,2,3,4,5,6\right)$$

where, AP was used to represent the relative light intensity of the six peaks (P1, P2, P3, P4, P5, P6) for fungal spores’ diffraction fingerprint. AV was used to represent the relative light intensity of the six valley points (V1, V2, V3, V4, V5, V6) for fungal spores’ diffraction fingerprint. C was used to represent the relative light intensity of the central area for fungal spores’ diffraction fingerprint.

### 2.6. Sample Division and Test Platform

In this study, a total of 600 diffraction fingerprint images of fungal spores were collected (200 for each disease spore), 70% of which were randomly selected for training and 30% for testing [34]. The Peak to Center ratio (PCR), Valley to Center ratio, and Peak to Valley ratio (PVR) features were extracted from each diffraction fingerprint image. A total of 18 features were extracted from each image. The classification experiment based on the diffraction fingerprint image characteristics of fungal spores was carried out in a MATLAB R2016b environment. The computer system configuration used was Win10 (64-bit), with a running memory of 16 G, and an Intel(R) Core (TM)i5-9400F CPU @ 2.90 GHz processor.

### 2.7. Evaluation Index of Classification Results

In addition, common classification performance metrics were used to evaluate classification models in this study. These classification performance metrics include accuracy, precision, recall, and comprehensive evaluation index F values (F1-Score). The calculation formulas are shown in formulas (8)–(11) [35].

(8) $$\mathrm{Accuracy}=\frac{\mathrm{TP}+\mathrm{TN}}{\mathrm{TP}+\mathrm{TN}+\mathrm{FP}+\mathrm{FN}}$$

(9) $$\mathrm{Pr}\mathrm{ecision}=\frac{\mathrm{TP}}{\mathrm{TP}+\mathrm{FP}}$$

(10) Recall=TPTP+FN

(11) F1-Score=2×Precision×RecallPrecision+Reacll

where TP is used to represent true positive, TN is used to represent true negative, FP is used to represent false positive, and FN is used to represent false negative.

### 2.8. Statistical Analysis Software

In this study, pathogenic spore diffraction was processed by MATLAB R2016b software. All algorithms were run in a MATLAB R2016b environment.

## 3. Results

### 3.1. Results and Analysis of Fungal Spore Diffraction Fingerprint Image

**Figure 5:**
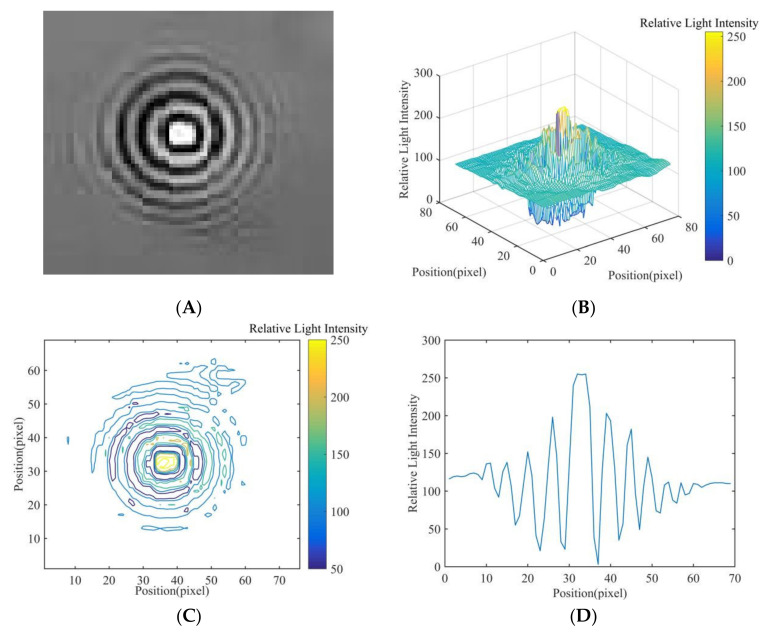
Results of *P. cubensis* spore fingerprint image processing, (**A**) diffraction image, (**B**) three-dimensional relative light intensity distribution, (**C**) two-dimensional relative light intensity distribution, and (**D**) relative light intensity distribution value.

**Figure 6:**
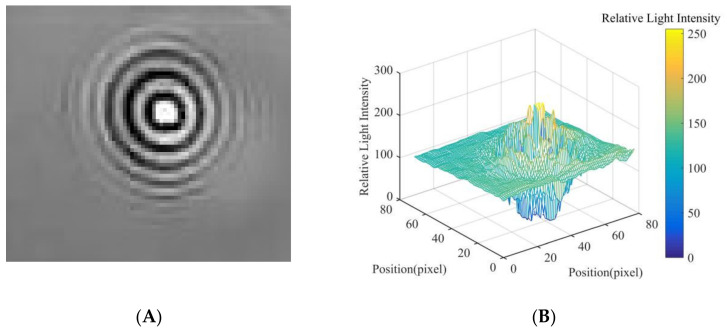

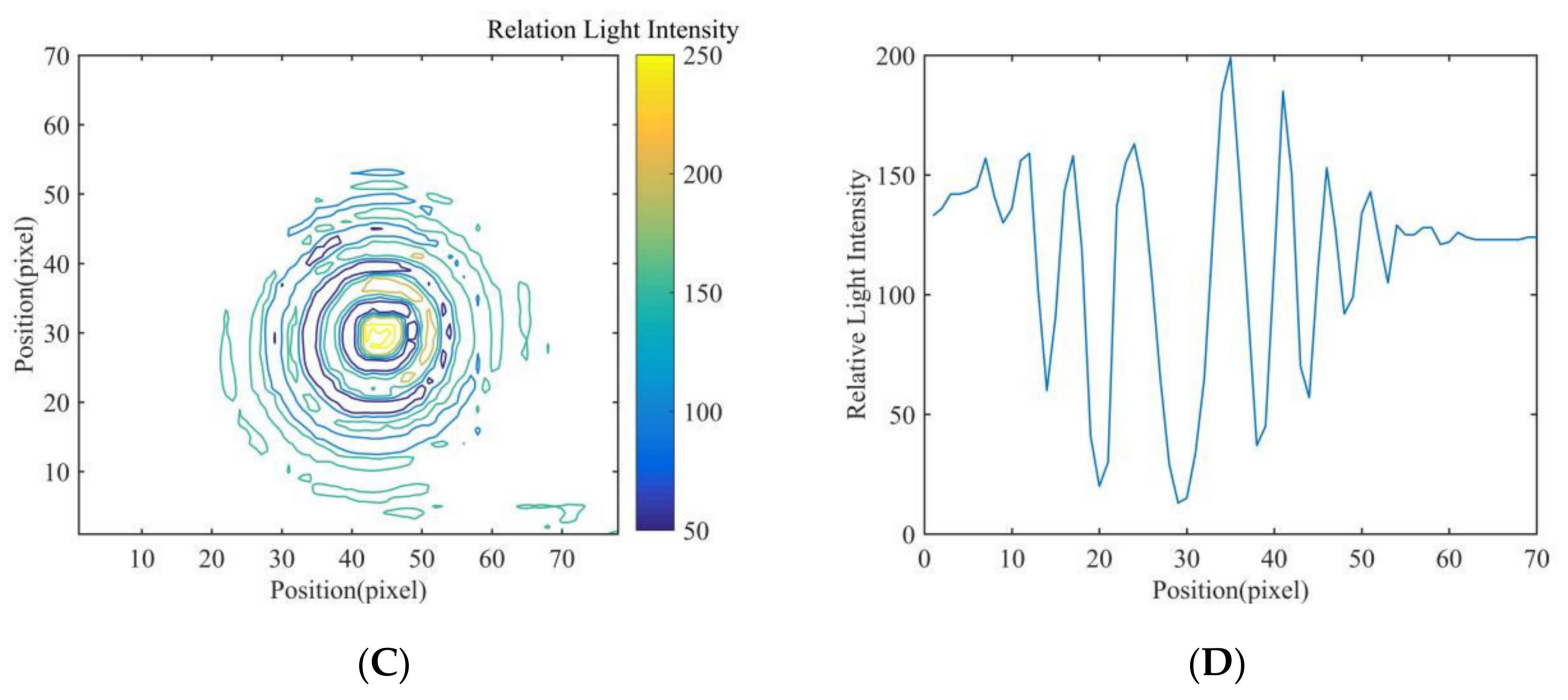
Results of *P. xanthii* spore fingerprint image processing, (**A**) diffraction image, (**B**) three-dimensional relative light intensity distribution, (**C**) two-dimensional relative light intensity distribution, and (**D**) relative light intensity distribution value.

**Figure 7:**
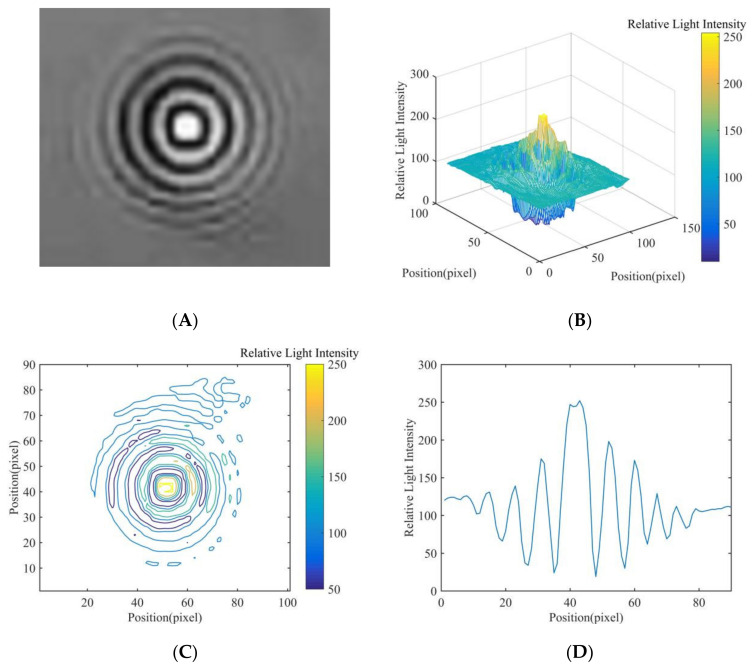
Results of *B. cinerea* spore fingerprint image processing, (**A**) diffraction image, (**B**) three-dimensional relative light intensity distribution, (**C**) two-dimensional relative light intensity distribution, and (**D**) relative light intensity distribution value.

In this study, in order to extract the diffraction fingerprint image features of the fungal spores, diffraction images of fungal spores were collected and pre-processed. The results are shown in Figure 5A, Figure 6A and Figure 7A. Based on this, the three-dimensional relative light intensity distribution (as shown in Figure 5B, Figure 6B and Figure 7B) and two-dimensional relative light intensity distribution (as shown in Figure 5C, Figure 6C and Figure 7C) of the diffraction fingerprint image of fungal spores were obtained. Figure 5B, Figure 6B and Figure 7B demonstrate that the relative light intensity value was the highest near the center of the diffracted fingerprint. The relative light intensity value of the background of the processed spore diffraction image was distributed around 100. The relative light intensity values of the diffraction images of *P. cubensis* spores and *P. xanthii* spores were distributed between 0 and 255. The relative light intensity values of the diffraction images of *B. cinerea* spores were distributed between 50 and 255. Figure 5C, Figure 6C and Figure 7C show that the relative light intensity in the region near the center of the diffraction fingerprint image of fungal spores was the largest. The main stripes of the fungal spores’ diffraction fingerprint image are more valuable, followed by the 6 stripes next to them. In addition, the spatial positions of the two-dimensional light intensity distribution of the spores’ diffraction images of the three kinds of airborne diseases were different. This may be related to the size and shape of the spores. To obtain the relative light intensity value of the characteristic fingerprint of the diffraction fingerprint image of fungal spores, a two-dimensional relative light intensity distribution image of the diffraction fingerprint image of the fungal spores was processed. The relative light intensity distribution value of each diffraction fringe was obtained. The results are shown in Figure 5D, Figure 6D and Figure 7D.

Then, the relative light intensity distribution values of the spores’ diffraction fingerprint characteristics of the three kinds of airborne diseases were statistically analyzed. The results are shown in Table 1. It can be seen from Table 1 that the relative light intensity value range of the central fringe of the *P. cubensis* spore diffraction fingerprint image was 243–275. The relative light intensity value range of the main bright fringe was 102–223. The relative light intensity value range of the main dark fringe was 8–106. The relative light intensity value range of the central fringe of the *P. xanthii* spore diffraction fingerprint image was 182–224. The relative light intensity value range of the main bright fringe was 131–195. The relative light intensity value range of the main dark fringe was 12–107. The relative light intensity value range of the central fringe of the *B. cinerea* spore diffraction fingerprint image was 253–286. The relative light intensity value range of the main bright fringe was 104–215. The relative light intensity value range of the main dark fringe was 14–83.

### 3.2. Analysis of Classification Results

#### 3.2.1. Evaluation Index 

**Figure 8:**
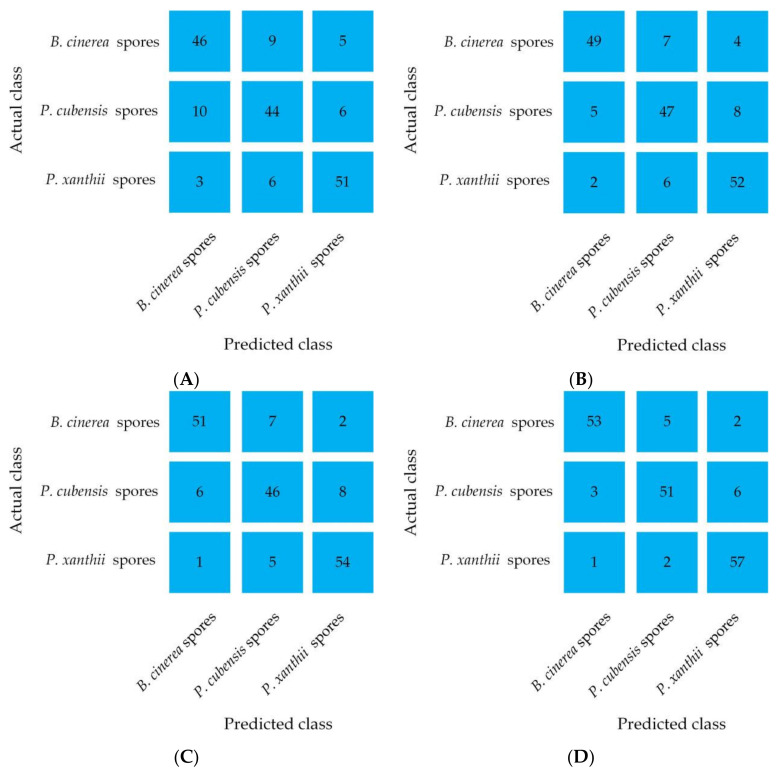
Confusion matrix of different classification models, (**A**) LR model, (**B**) KNN model, (**C**) RF model, and (**D**) SVM model.

Instability caused by possible sample size bias was considered when classifying pathogenic spores’ diffraction fingerprint images from the three diseases using the LR model, and a one-to-one classification method was used. The LR algorithm chooses L2 as the regular term. When the KNN model was used to classify pathogenic spores’ diffraction fingerprint images, 70% of the training set was reclassified considering the number of samples, and the K value of the KNN algorithm was estimated by 10-fold cross-validation. When using the RF model to classify pathogenic spores’ diffraction fingerprint images, in order to achieve the purpose of extracting equal samples, the adopted sampling method was put back into the sampling. SVM algorithm uses a Gaussian kernel function and adopts a one-to-one classification method. In this study, a confusion matrix was used to evaluate the performance of the classifier. As shown in Figure 8, each row of the matrix represents the actual category for the sample, and each column represents the predicted category for the sample. The numbers on the diagonal of the matrix represent the number of samples correctly identified for each category [36]. The results of the LR classification model (Figure 8) show that 46, 44, and 51 samples of *B. cinerea* spores, *P. cubensis* spores, and *P. xanthii* spores were correctly identified. Among them, 9 samples of *B. cinerea* spores were predicted to be *P. cubensis* spores, and 5 samples of *B. cinerea* spores were predicted to be *P. xanthii* spores. For the *P. cubensis* spores, 10 samples were predicted to be *B. cinerea* spores, and 6 samples were predicted to be *P. xanthii* spores. For the *P. xanthii* spores, 3 samples were predicted to be *B. cinerea* spores, and 6 samples were predicted to be *P. cubensis* spores. The results of the KNN classification model show that 7 samples of *B. cinerea* spores were predicted to be *P. cubensis* spores, and 4 samples of *B. cinerea* spores were predicted to be *P. xanthii* spores. For the *P. cubensis* spores, 5 samples were predicted to be *B. cinerea* spores, and 8 samples were predicted to be *P. xanthii* spores. For the *P. xanthii* spores, 2 samples were predicted to be *B. cinerea* spores, and 6 samples were predicted to be *P. Cubensis* spores. The results of the RF classification model show that 7 samples of *B. cinerea* spores were predicted to be *P. cubensis* spores, and 2 samples of *B. cinerea* spores were predicted to be *P. xanthii* spores. For the *P. cubensis* spores, 6 samples were predicted to be *B. cinerea* spores, and 8 samples were predicted to be *P. xanthii* spores. For the *P. xanthii* spores, 1 sample was predicted to be *B. cinerea* spores, and 5 samples were predicted to be *P. cubensis* spores. The results of the SVM classification model show that 5 samples of *B. cinerea* spores were predicted to be *P. cubensis* spores, and 2 samples of *B. cinerea* spores were predicted to be *P. xanthii* spores. For the *P. cubensis* spores, 3 samples were predicted to be *B. cinerea* spores, and 6 samples were predicted to be *P. xanthii* spores. For the *P. xanthii* spores, 1 sample was predicted to be *B. cinerea* spores, and 2 samples were predicted to be *P. cubensis* spores.

The classification results of different classification models are shown in Table 2.

#### 3.2.2. Classification Results of Different Models

In this study, the Peak to Center ratio (PCR), Valley to Center ratio, and Peak to Valley ratio (PVR) were extracted from the diffraction fingerprint images of airborne disease spores. The classification models of LR, KNN, RF, and SVM were established based on the features of the airborne disease spore’s diffraction fingerprint. The accuracy, precision, recall, and F1-Score of three kinds of fungal spores from greenhouse crops, classified by LR, KNN, RF, and SVM models were compared and analyzed. The results are shown in Table 3 and Table 4.

As shown in Table 3, with regards to the three kinds of fungal spores from greenhouse crops, the SVM model has higher accuracy, precision, recall, and F1-Score than the LR, KNN, and RF classification models. For *B. cinerea* spores, the accuracy, precision, recall, and F1-Score of the SVM model were 93.6%, 92.98%, 88.33%, and 90.6%, respectively. For *P. cubensis* spores, the accuracy, precision, recall, and F1-Score of the SVM model were 90.96%, 87.93%, 85%, and 86.44%, respectively. For *P. xanthii* spores, the accuracy, precision, recall, and F1-Score of the SVM model were 93.60%, 87.69%, 95%, and 91.2%, respectively.

As shown in Table 4, the SVM model has a better overall classification performance compared to the LR, KNN, and RF models. The average recognition accuracy rates of the three kinds of fungal spores from greenhouse crops under the SVM model were 92.72%, while the accuracy rates of the LR, KNN, and RF models were 84.97%, 87.44%, and 88.72%, respectively. As a comprehensive evaluation index, the F1-Score represents the overall performance of the classifier. The F1-Score value of the SVM model was higher, and the overall average value reached 89.41%, which were 11.12%, 7.18%, and 5.57% higher than the LR model, KNN model and RF model, respectively, showing that the SVM model has a good comprehensive performance.

## 4. Discussion

Tomatoes and cucumbers, as the most widely planted important vegetables in greenhouses, have high economic benefits. Due to the environmental conditions in greenhouses, cucumber downy mildew, powdery mildew, and tomato gray mold are prone to occur and these diseases can easily cause crop yield losses. Therefore, it is of great importance to detect and control the airborne disease spores of greenhouse crops to stabilize and increase crop yield. Traditional methods rely on agronomists manually checking the plant disease symptoms or visible signs of pathogens with the naked eye or professional analysts performing physiological and biochemical analyses including molecular, serological, and deoxyribose nucleic acid. The method of physiological and biochemical analysis is time-consuming and labor-intensive, and specific operating environments, as well as a high level of analyst expertise and operating skills, are needed to obtain reliable diagnosis results. In addition, these conventional methods are inadequate for detecting diseases in the latent period, because the symptoms of the latent period are invisible to the naked eye [37,38].

Early prediction and control of diseases can be realized by using spore catchers to capture and detect spores in the air [27,28,29,30]. Measuring and identifying spores is a standard method for describing fungi classification and with manual microscopic analyses, the number of spores that can be measured and their morphological traits can be observed. However, due to the small size and the large number of fungal spores, and the small field of view of the microscope, large counting errors can occur easily [33]. Lens-less CMOS image sensors are favored by related researchers due to their large imaging field of view and low cost [39]. To overcome this challenge, we present a rapid detection method for fungal spores from greenhouse crops based on CMOS image sensors and diffraction fingerprint feature processing. Diffraction refers to the phenomenon that light encounters opaque or transparent obstacles or micropores (narrow slits) in the propagation path, bypasses the obstacles, and deviates from the straight line. Under the appropriate circumstances, any wave has the inherent properties of diffraction and the degree of wave diffraction varies in different situations. If the obstacle has multiple densely distributed pores, it will cause a more complicated diffraction intensity distribution pattern. The size, shape, and surface texture characteristics of different disease spores are varied [40]. The light intensity distribution of light and dark stripes generated during diffraction can reflect characteristic information of disease spores.

Pathogenic spores are mainly composed of a cell wall and cytoplasm, and pathogenic spores absorb and reflect light differently. Therefore, a visible and near-infrared hyperspectral imaging (HSI) system can be used to measure the growth of fungi in the wavelength range of 400 to 1000 nm and find and identify fungal infections [41,42]. For example, Lu et al. [43] collected spectral data of *Aspergillus parasiticus*, *Aspergillus flavus*, *Aspergillus glaucus*, *Aspergillus niger*, and *Penicillium* sp. and established a corresponding multispectral classification model. Using the SPA-SVM method to analyze the HSI images of the five fungi grown for one day, the five fungi could be well distinguished. This method requires a certain period of time to cultivate pathogenic bacteria, needs to be carried out in a laboratory, which is inconvenient to operate and has a certain lag in disease detection, prevention, and control [44]. In addition, each pathogenic spore has its shape characteristics, including perimeter, surface area, aspect ratio, and roundness. Using these features, the method of image processing can identify and classify the spores of different pathogens [45]. For example, Yang et al. [46] used a spore capture device to capture rice blast fungus spores in the air and collected microscopic images of rice blast fungus spores. The shape features such as area, perimeter, ellipticity, and complexity of the spores of rice blast were extracted, as well as texture features such as entropy, uniformity, and contrast. Then a decision tree model was established to detect the spores of rice blast. Although spore capturing instruments and microscopic image processing methods can detect pathogenic bacteria spores, a microscope is required, and the traditional microscope has a small field of view and cannot meet the needs of the actual situation [27,28,46]. Relevant studies have shown that when the number of spores is small, the accuracy of manual identification may be higher. However, when the number is large, the advantages of machine recognition are reflected, which can save a lot of time and manpower [47]. In this study, a rapid detection method of fungal spores in greenhouse crops based on CMOS image sensors and diffraction fingerprint feature processing was proposed. Pathogenic spores do not need to be cultured and they can be detected more quickly. In addition, this study uses a CMOS image sensor to directly collect the diffractive fingerprint images of pathogenic spores. Due to the large imaging field of view of the lens-less CMOS image sensor, more pathogenic spores’ images can be captured. The method proposed in this study has slightly higher accuracy in identifying disease spores than the method of microscopic image processing of disease spores [28]. In addition, the method proposed in this article is easy to use and cheaper than traditional microscopic imaging systems. It is more conducive to actual promotion and application [27,29,30].

## 5. Conclusions

In this study, to achieve rapid detection of spores of airborne diseases in greenhouse crops, a rapid detection method of fungal spores from greenhouse crops based on CMOS image sensors and diffraction fingerprint feature processing is proposed. The diffraction fingerprint image acquisition system for fungal spores in greenhouse crops was built. Diffraction fingerprint images of three kinds of disease spores were collected. The diffraction fingerprint feature of disease spores was extracted and the classification model was established. The SVM model had higher accuracy, precision, recall, and F1-Score than the LR, KNN, and RF classification models. For *B. cinerea* spores, the accuracy, precision, recall, and F1-Score of the SVM model were 93.6%, 92.98%, 88.33%, and 90.6%, respectively. For *P. cubensis* spores, the accuracy, precision, recall, and F1-Score of the SVM model were 90.96%, 87.93%, 85%, and 86.44%, respectively. For *P. xanthii* spores, the accuracy, precision, recall, and F1-Score of the SVM model were 93.60%, 87.69%, 95%, and 91.2%, respectively. The method proposed in this study can be used for remote identification of three fungal spores and it is a low-cost and portable system.

This research mainly focused on the spores of three pathogens. However, there may be other biological and non-biological particles in greenhouses. In addition, in this study, the collection of pathogenic spores needed the assistance of a portable spore trap. Although the portable spore trap can catch spores, it cannot separate and enrich pathogenic spores. Therefore, it is suggested that in the future, more research on the influence of biological and non-biological particles on the experiment, as well as the separation and enrichment methods of spores be considered.

## Figures and Tables

**Table 1:** Statistical results of the relative light intensity distribution of diffraction fingerprints of three kinds of fungal spores.

Feature		Relative Light Intensity Distribution Value		
		*P. cubensis* Spore	*P. Xanthii* Spore	*B. cinerea* Spore
Center	C	243–275	182–224	253–286
Main bright fringe	P1 and P6	102–154	131–164	104–153
	P2 and P5	150–183	141–173	133–206
	P3 and P4	184–223	152–195	172–215
Main dark fringe	V1 and V6	53–106	49–107	61–83
	V2 and V5	26–51	23–63	21–52
	V3 and V4	8–33	12–39	14–37

**Table 2:** Classification results of different classification models.

Species	Basic Indicators															
	LR				KNN				RF				SVM			
	TP	TN	FP	FN	TP	TN	FP	FN	TP	TN	FP	FN	TP	TN	FP	FN
*B. cinerea* spores	46	95	13	14	49	99	7	11	51	100	7	9	53	108	4	7
*P. cubensis* spores	44	97	15	16	47	101	13	13	46	105	12	14	51	110	7	9
*P. xanthii* spores	51	90	11	9	52	96	12	8	54	97	10	6	57	104	8	3

**Table 3:** Comparative analysis of different classification models (%).

Species	Classification Results															
	Accuracy				Precision				Recall				F1-Score			
	LR	KNN	RF	SVM	LR	KNN	RF	SVM	LR	KNN	RF	SVM	LR	KNN	RF	SVM
*B. cinerea* spores	83.93	89.16	90.42	93.60	77.97	87.50	87.93	92.98	77.67	81.67	85.00	88.33	77.31	84.48	86.44	90.60
*P. cubensis* spores	81.98	85.06	85.31	90.96	74.58	78.33	79.31	87.93	73.33	78.33	76.67	85.00	73.95	78.33	77.97	86.44
*P. xanthii* spores	87.58	88.10	90.42	93.60	82.26	81.25	84.38	87.69	85.00	86.67	90.00	95.00	83.61	83.87	87.10	91.20

**Table 4:** The overall average recognition ability comparisons of different classification models (%).

Indexes	Classification Model			
	LR	KNN	RF	SVM
Accuracy	84.97	87.44	88.72	92.72
Precision	78.27	82.36	83.87	89.53
Recall	78.67	82.22	83.89	89.44
F1-Score	78.29	82.23	83.84	89.41

## Data Availability

Not applicable.
